# Modified SHI medium supports growth of a disease‐state subgingival polymicrobial community in vitro

**DOI:** 10.1111/omi.12323

**Published:** 2020-12-03

**Authors:** Eleanor I. Lamont, Archita Gadkari, Kristopher A. Kerns, Thao T. To, Diane Daubert, Georgios Kotsakis, Batbileg Bor, Xuesong He, Jeffrey S. McLean

**Affiliations:** ^1^ Department of Periodontics University of Washington Seattle WA USA; ^2^ Department of Periodontics University of Texas Health Science Center San Antonio TX USA; ^3^ Department of Microbiology The Forsyth Institute Cambridge MA USA; ^4^ Department of Oral Medicine, Infection and Immunity Harvard School of Dental Medicine Boston MA USA

**Keywords:** In vitro biofilm model, periodontitis, SHI‐media, subgingival microbiota

## Abstract

Developing a laboratory model of oral polymicrobial communities is essential for in vitro studies of the transition from healthy to diseased oral plaque. SHI medium is an enriched growth medium capable of supporting in vitro biofilms with similar diversity to healthy supragingival inocula; however, this medium does not maintain the diversity of gram‐negative bacteria more associated with subgingival plaque. Here, we systematically modified SHI medium components to investigate the impacts of varying nutrients and develop a medium capable of supporting a specific disease‐state subgingival community. A diseased subgingival plaque sample was inoculated in SHI medium with increasing concentrations of sucrose (0%, 0.1%, 0.5%), fetal bovine serum (FBS) (0%, 10%, 20%, 30%, 50%), and mucin (0.1, 2.5, 8.0 g/L) and grown for 48 hrs, then the 16S rRNA profiles of the resulting biofilms were examined. In total, these conditions were able to capture 89 of the 119 species and 43 of the 51 genera found in the subgingival inoculum. Interestingly, biofilms grown in high sucrose media, although dominated by acidogenic Firmicutes with a low final pH, contained several uncultured taxa from the genus *Treponema*, information that may aid culturing these periodontitis‐associated fastidious organisms. Biofilms grown in a modified medium (here named subSHI‐v1 medium) with 0.1% sucrose and 10% FBS had a high diversity closest to the inoculum and maintained greater proportions of many gram‐negative species of interest from the subgingival periodontal pocket (including members of the genera *Prevotella* and *Treponema*, and the Candidate Phyla Radiation phylum Saccharibacteria), and therefore best represented the disease community.

## 1 INTRODUCTION

Subgingival oral plaque communities play an essential role in the initiation and progression of periodontal disease. It is now appreciated that disease progression is a shift of the microbial community from health to a dysbiotic disease state, triggering the host inflammatory response and ultimately tissue and bone loss (Hajishengallis, 2014; Lamont et al., 2018). Single species studies on model periopathogens and their interactions with the host through in vitro experiments and animal models have revealed many important insights (Mountcastle et al., 2020). Due to their complexity, studies using diverse multi‐species communities have progressed to a lesser extent; however, they are essential for understanding oral diseases such as gingivitis and periodontitis (Edlund et al., 2013; Mountcastle et al., 2020).

Early in vitro models of subgingival plaque bacteria were constructed of communities containing only a handful of species (e.g., Ammann et al., 2012; Sánchez et al., 2011; Sissons, 1997; Soares et al., 2015). The organisms chosen were considered important to initiating or sustaining disease and, perhaps more importantly, could be cultured in the lab. It is now clear, however, that progression to a disease state is caused by more than a few species, and to understand periodontal disease the community as a whole needs to be addressed (Dzink et al., 1985; Marsh, 2003; Socransky et al., 1998; Theilade, 1986). Next‐generation sequencing methods, specifically 16S amplicon sequencing, allow multi‐species samples to be quickly and easily characterized, and research applying this technique has progressed to enable rapid development of more representative complex polymicrobial in vitro biofilms (McLean, 2014). The main challenge of this approach is to generate in vitro models representative of an in vivo community both in taxonomic proportions and abundances. To address this, different methods of growing plaque‐derived in vitro biofilms are being developed, with various conditions such as medium composition, inoculum source, and growth time investigated (e.g., Baraniya et al., 2020; Cieplik et al., 2019; Velsko & Shaddox, 2018).

SHI medium, an enriched growth medium originally developed to support supragingival bacterial communities, can maintain in vitro biofilms representative of a diverse inoculum with high reproducibility, capturing 60%–80% of the taxonomic abundance from the original community (Edlund et al., 2013; Tian et al., 2010). Diverse models such as these have many benefits over individual monocultures and limited multi‐species models using laboratory strains, and this approach has allowed the profiling of activity and function of the community using parallel omic techniques (metatranscriptomics and metabolomics) (Edlund et al., 2015, 2018). These in vitro communities have also shown to include organisms otherwise recalcitrant to traditional culturing techniques. *Nanosynbacter lyticus* stain TM7x of the Phylum Saccharibacteria (Formerly TM7), a member of the Candidate Phyla Radiation (CPR), was the first cultured representative of the CPR and was initially isolated from saliva‐derived microbial community cultured in SHI medium, validating the utility of this medium in sustaining a highly diverse *in vitro* microbiome (Edlund et al., 2013; He et al., 2015).

Saliva‐derived, healthy supragingival communities with high abundances of gram‐positive, saccharolytic organisms grow well in SHI medium, but this medium was not designed to support the proteolytic, gram‐negative microbiota more associated with disease‐state subgingival plaque. In this study, we systematically modified the well‐characterized SHI medium to investigate how changes in key components can enable the growth of subgingival‐associated species. In addition to developing a formulation for diverse subgingival in vitro communities representative of a diseased state, the knowledge of which medium alterations could impact the community composition will help understand the shift between healthy and disease states and is of interest to understand clinical interventions that may be beneficial for periodontitis patients.

## 2 METHODS

### 2.1 Plaque and saliva collection

Subgingival plaque was sampled from an ex‐smoker African American female with Stage 3, grade C periodontitis (Papapanou et al., 2018). African American ethnicity and ex‐smoker status represent a subgroup with high prevalence of periodontitis among US adults ≥30 years (Eke et al., 2018), and thus the microbial community is of high significance to model in vitro. This particular sample was utilized as the plaque inoculum for all in vitro biofilms due to its high biomass, which facilitated replicate and repeated examinations. Plaque was collected using a sterile curette and stored in 500 µl Anaerobic Transport Medium (ATM) (Anaerobe Systems CAT# AS‐916) with added 16% glycerol, vortexed to homogenize the sample, then further diluted in 5 ml ATM/glycerol, aliquoted, and stored at −80°C. Unstimulated saliva was collected from four healthy subjects, age 25–50 years and was used to coat plate surfaces to enhance bacterial attachment and biofilm growth. Subjects were asked to refrain from brushing the morning of collection and instructed to spit saliva directly into the saliva collection tube. Saliva samples were pooled and centrifuged at 2,600 *g* for 10 min to spin down large debris and eukaryotic cells, and the supernatant was collected as pooled saliva and stored at 4°C (Tian et al., 2010). Patient consent was obtained to participate in this study, and the Institutional Review Board confirmed ethical approval for all saliva and subgingival plaque collection protocols and research under the approved protocol (University of Washington IRB No. 51195).

### 2.2 SHI medium variations

The original SHI medium is defined in Tian et al. (2010) with the following composition: 10 g/L proteose peptone (Fisher, BP1420‐500); 5.0 g/L trypticase peptone (Fischer, BP 1421‐500); 5.0 g/L yeast extract (BD Bacto, 212750); 2.5 g/L KCl (Fisher, P217‐500); 5 mg/L hemin (Sigma, 51280); 1 mg/L Vitamin K (Sigma, M5625‐25G); 0.06 g/L urea (Fisher, U15‐3), 0.174 g/L arginine (Fisher, BP370‐100); 2.5 g/L mucin (Sigma, M1778‐10G); 10 mg/L *N* ‐acetylmuramic acid (Sigma, A3008‐100MG); 5% sheep blood (Colorado serum company) and 0.5% sucrose (Sigma, S7903).

SHI medium was modified with increasing concentrations of sucrose, heat‐inactivated fetal bovine serum (FBS), and mucin. Sucrose, a carbon source for supragingival bacteria in vivo and at a concentration of 0.5% in original SHI medium, was tested at 0%, 0.1%, 0.5%, and 0.8%. Sucrose was used instead of glucose as it increased recovery of streptococci in the original SHI medium (Tian et al., 2010). FBS (Gibco, A3160501), a common medium supplement containing similar growth factors to gingival crevicular fluid (GCF), is not included in original SHI medium. Here, the FBS concentrations 0%, 10%, 20%, 30%, and 50% were tested. Mucin, another supragingival carbon source at a concentration of 2.5g/L in original SHI medium, was tested at 0, 0.1, 2.5, and 8 g/L. Combinations of medium conditions are listed in Table 1; in total 15 media variations were tested. An initial survey was conducted sequencing single replicate in vitro biofilms. Based on this preliminary analysis, the most promising conditions were replicated and further media variations were tested in an iterative process to refine the model.

**TABLE 1:** Media conditions with number of replicates, average final pH, average amplicon strain variants (ASVs), and average species numbers

Condition			Replicates	Average Final pH ± *SD*	Average ASVs ± *SD*	Average species ± *SD*
% FBS	% sucrose	g/L mucin				
0	0	2.5	1	7.2	64	50
0	0.1	2.5	5	6.88 ± 0.38	51.8 ± 12.24	43.8 ± 8.04
0	0.5	2.5	3	4.53 ± 0.15	46.7 ± 10.26	41.3 ± 8.39
0	0.8	2.5	2	4.65 ± 0.07	51 ± 12.73	44.5 ± 9.19
10	0	2.5	3	7.23 ± 0.12	57.33 ± 2.08	44 ± 2.65
**10**	**0.1**	**2.5**	**3**	**6.3 ± 0.70**	**59.3 ± 20.98**	**49.3 ± 16.65**
10	0.5	2.5	2	4.5 ± 0.14	38.5 ± 4.95	35.5 ± 6.36
10	0.8	2.5	2	4.7 ± 0	41 ± 8.49	38 ± 8.49
20	0	2.5	3	6.73 ± 0.12	63 ± 9.64	46.7 ± 5.51
30	0.1	2.5	1	6.1	40	35
50	0.1	2.5	1	5.6	28	24
10	0	0	3	7.47 ± 0.06	57.33 ± 2.89	45.67 ± 2.31
0	0.1	0.1	1	7.1	55	44
30	0.1	0.1	3	5.23 ± 0.06	24 ± 4.36	22.33 ± 3.79
0	0.1	8.0	1	6.4	49	43

Note

subSHI‐v1 medium is marked in bold.

Abbreviation: *SD*, standard deviation.

### 2.3 Growth of in vitro biofilms

Biofilms were grown following the methods of Edlund et al. (2013, 2015, 2018). Briefly, SHI‐medium was reduced in an anaerobic chamber (5% H_2_, 5% CO_2_, 90% N_2_) for 3–4 hr and the initial pH, ranging from 7.0–7.3, was measured prior to inoculation. To produce a saliva pellicle and aid biofilm formation, 24‐well plates were precoated with 200µl pooled saliva, dried in an incubator at 37°C for 1 hr, and UV sterilized for 1 hr. Reduced SHI medium (1.9 ml) was added to each well and inoculated with 20 µl of the subgingival plaque inoculum. For each medium condition an uninoculated media control was incubated on the plate and processed with the inoculated samples. This served as a negative control for any potential bacterial DNA amplified and sequenced from the saliva pellicle. Plates were then incubated in an anaerobic chamber at 37°C for 48 hrs. After incubation, the final pH of the medium was measured without disturbing the biofilm, then the biofilms were resuspended and collected in a 2 ml tube. Tubes were centrifuged to pellet the biofilm and supernatant was removed. Pellets were stored at −80°C.

### 2.4 DNA extraction and 16S sequencing

Genomic DNA from biofilm pellets were extracted using the QIAamp DNA Microbiome Kit (Qiagen, CAT# 51704) and further purified and concentrated using DNA Clean and Concentrator Kit (Zymo Research, CAT# R1016) according to the manufacturer's protocols. DNA concentration was determined with Qubit Broad Range dsDNA assay (Thermo Fisher Scientific), and samples were stored at −80°C.

16S rRNA gene sequencing was performed for all samples on an Illumina MiSeq platform. Samples were sequenced on two sequencing runs. Each run included a negative kit control, a no template library preparation control, and a Zymogenetics bacterial community standard (Zymo Research, CAT# D6310) positive control. The plaque inoculum was sequenced twice to identify any possible variability between the two runs.

Library preparation was performed as follows: The V3‐V4 variable region of the 16S rRNA gene was amplified using gene‐specific primers with Illumina adapter overhang sequences (5′‐TCGTCGGCAGCGTCAGATGTGTATAAGAGACAGCCTACGGGNGGCWGCAG‐3′ and 5′‐GTCTCGTGGGCTCGGAGATGTGTATAAGAGACAGGACTACHVGGGTATCTAATCC‐3′). Each reaction mixture contained 2.5 µl of genomic DNA, 5 µl of each 1 µM primer, and 12.5 µl of HiFi HotStart ReadyMix (KAPA, CAT# KK2602). Amplicon PCR was carried out with the following parameters: 95°C for 3 min, 35 cycles at [95°C for 30 s, 55°C for 30 s, 72°C for 30 s], followed by extension at 72°C for 5 min. PCR products were verified by gel electrophoresis (1% agarose gel) and cleaned with AMPure XP beads (Beckman Coulter, A63881). Amplicons were indexed using the Nextera XT Index Kit V2 set A and set D (Illumina) and purified again with AMPure XP beads to remove low molecular weight primers and primer‐dimer sequences. SequalPrep Normalization Kit (Invitrogen, A10510‐01) was used to normalize samples to a concentration of 1–2 ng/µL. Samples were pooled into a single library and checked for DNA quality and quantity using a TapeStation 4200 High Sensitivity D1000 assay (Agilent Technologies) and Qubit High Sensitivity dsDNA assay (Thermo Fisher Scientific), respectively. The final pooled library was loaded on to an Illumina MiSeq with 10% PhiX spike, which served as an internal control to balance for possible low diversity and base bias present in the 16S amplicon samples, was run for 301 cycles (2 × 301 bp), and generated an average of 106,000 reads per sample.

### 2.5 16S data processing and bioinformatics

Demultiplexed paired‐end sequences were downloaded from the Illumina MiSeq platform, imported to QIIME2 (v2019.10), and trimmed and denoised using the DADA2 package (Callahan et al., 2016). Forward reads were trimmed by 15 nucleotides (nt) from the 5′ end and 11 nt from the 3′ end; reverse reads were trimmed by 15 nt from the 5′ end and 56 nt from the 3′ end. Taxonomic assignment to classify the amplicon sequence variants (ASVs) was performed using the feature‐classifier suite trained on the Human Oral Microbiome Database (HOMD v15.1) (Escapa et al., 2018). 16S sequence data have been deposited to the Genbank database under Bioproject number PRJNA6633916 and raw read counts for each sample are provided in Table S1. The .biom file generated in QIIME2 was imported to RStudio v1.2.1335 for further processing.

Commonly found kit contaminants as well as taxa enriched in uninoculated media controls but absent from the plaque inoculum were removed in RStudio using Phyloseq v1.30.0 (McMurdie and Holmes, 2013). The following taxonomic assignments were removed: Unassigned, *Delftia, Yersinia, Listeria, Mogibacterium, Acinetobacter, Jeogalicoccus, Pseudomonas, Pseudoramibacter, Arthrospira platensis, Bacillus subtilis, Variovorax paradoxus, Escherichia coli, Pseudomonas aeruginosa,* and *Pseudomonas fluorescens*. Additionally, two ASVs (classified to *Streptococcus oralis* and *Veillonella dispar* species) found at high abundance in all samples and negative controls were removed.

Analyses, including Alpha and Beta diversity, were performed in RStudio with the packages Phyloseq, ggplot2 v3.3.0 (Wickham, 2016), and ampvis2 v.2.5.9 (Andersen et al., 2018). Alpha diversity metrics (Shannon, Simpson, and Observed) were calculated with data rarefied to 15,141 sampling depth, the lowest sequence number found in a sample; all other analyses were performed on unrarefied data. Venn diagrams were produced with InteractiVenn (Heberle et al., 2015) and pie charts were produced with Krona (Ondov et al., 2011). For comparison, replicates were combined and DNA concentration, pH values, and taxonomic abundances were expressed as mean ± standard deviation (*SD*) for each medium condition. When appropriate, data were analyzed with the non‐parametric Wilcoxon test where *p* < .05 was considered statistically significant. Normality could not be assumed, and statistical comparisons could not be performed for several conditions with small sample sizes.

16S rRNA gene sequencing revealed several samples with enriched growth of Saccharibacteria phylum G3 group member HMT‐351, provisionally named “Ca. *Nanosyncoccus nanoralicus”* (McLean et al., 2018, 2020), that is likely an epibiont parasite on a bacterial host similar to strain TM7x (He et al., 2015). To identify the potential host of this bacterium, samples were divided into a group of biofilms with G3 HMT‐351 present and a group with G3 HMT‐351 absent, and the relative abundance of each species was averaged within each group. Onesided non‐parametric Wilcoxon tests were performed to determine which organisms were significantly (*p* < .05) enriched in biofilms containing G3 HMT‐351.

### 2.6 Whole genome sequencing

Whole genome shotgun (WGS) sequencing was performed on two samples enriched in “Ca. *N. nanoralicus”* G3 HMT‐351 (grown in the medium condition 10% FBS, 2.5 g/L mucin, 0.1% sucrose) at the University of Washington's Northwest Genomics Center (NWGC) to extract the genome of this strain. Samples were prepared with the PCR‐free KAPA HyperPrep Kit (Roche, Cat# 07962355001) and sequenced on an Illumina NovaSeq 6000 platform with a 300 cycle S Prime flow cell, generating 10 million reads per sample. Demultiplexed paired end reads were imported into KBase v2.1.4 (Arkin et al., 2018), the reads of both samples were combined into one library using Merge Reads Libraries v1.01, trimmed with Trimmomatic v0.36, and assembled with metaSPAdes v3.13.0 (into 4,795 contigs). Within Geneious v11, the Mauve plugin was used to map all assembled community contigs to a recently published genome of “Ca. *N. nanoralicus”* G3 HMT‐351 (McLean et al., 2020), generating a new set of 88 contigs. All reads were then mapped to the new set of contigs. Existing contigs and mapped reads were de novo assembled resulting in a partial genome of 39 contigs. The partial genome has been submitted to the Genbank database under Bioproject number PRJNA6633916.

## 3 RESULTS

### 3.1 Composition of plaque inoculum

To date, many studies seeking to reproduce oral plaque communities in vitro have relied on pooled plaque or pooled saliva from multiple subjects as the source inoculum (Baraniya et al., 2020; Edlund et al., 2013; Hope & Wilson, 2006). While a pooled inoculum can be beneficial, increasing diversity of models, it may ultimately lose details specific to the source sample and cannot address the variability found between oral sites. Periodontitis is a site‐specific disease that is noted for its higher microbial diversity than in health, and there can be significant taxonomic variability among patients and even between periodontal pockets of the same person (Mark Welch et al., 2019; Moore et al., 1984). In order to study the bacterial community that comprises a particular disease site, it is important to capture the diversity and composition of unique site‐specific microbiomes. Therefore, the current study used the same subgingival, aggressive periodontitis plaque sample for all in vitro biofilms.

**FIGURE 1:**
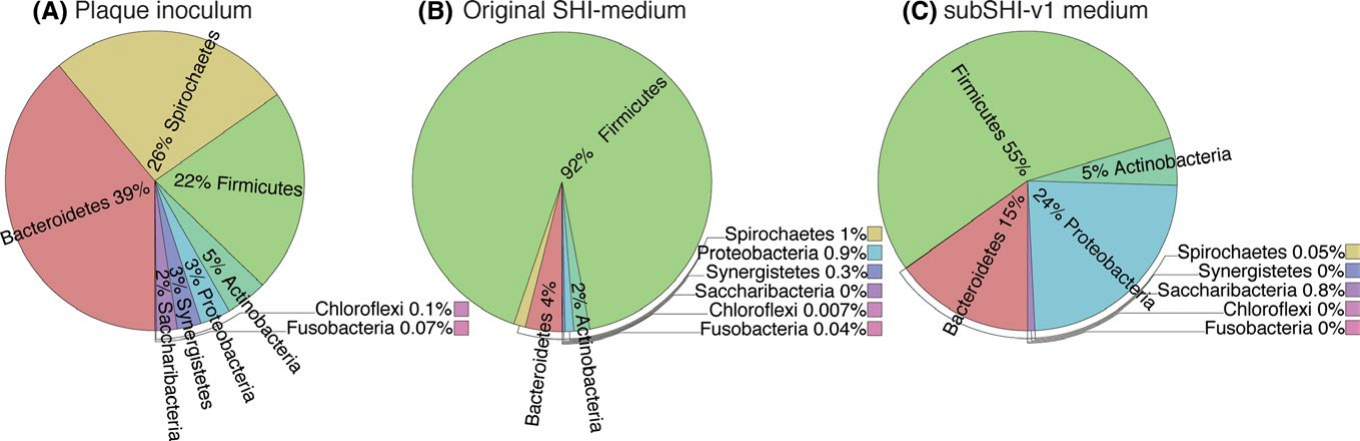
Average phylum level community profiles of (A) the plaque inoculum, biofilms grown in (B) original SHI medium (0.5% sucrose, 0% FBS, 2.5g/L mucin), and (C) subSHI‐v1 medium (0.1% sucrose, 10% FBS, 2.5g/L mucin) [Colour figure can be viewed at wileyonlinelibrary.com]

This inoculum consisted of nine phyla, 51 genera, 119 species, and 162 unique ASVs. Three major phyla—Bacteroidetes, Spirochaetes, and Firmicutes—dominated the sample, with smaller percentages of Actinobacteria, Proteobacteria, Synergistetes, Saccharibacteria, Chloroflexi, and Fusobacteria (Figure 1a). All of these phyla are commonly found in subgingival periodontitis samples at varying proportions (Armitage, 2010; Baraniya et al., 2020; Fernandez y Mostajo et al., 2017; Thompson et al., 2015). We do note that our inoculum was distinct from general descriptions of periodontitis communities. *Fusobacterium* is a common genus found in both health and disease communities (Colombo & Tanner, 2019) and its relative abundance can be over 30% of a periodontitis sample (Baraniya et al., 2020; Fernandez y Mostajo et al., 2017). However, just one species of *Fusobacterium* was present in the current inoculum, making up only 0.07% of the community. Of the classically described “red complex” bacteria (*Porphyromonas gingivalis, Tannerella. forsythia, Treponema. denticola*) often present in disease sites, this inoculum contained only *T. forsythia* and *T. denticola*. Within the *Porphyromonas,* only *P. endodontalis* was present (comprising 11% of the community); *P. gingivalis* was absent. This inoculum also contained a higher abundance and diversity of *Treponema* (19 species‐level taxa; 26% of the community) compared to pooled plaque samples described in the literature (Baraniya et al., 2020; Fernandez y Mostajo et al., 2017).

### 3.2 Sucrose variations

**FIGURE 2:**
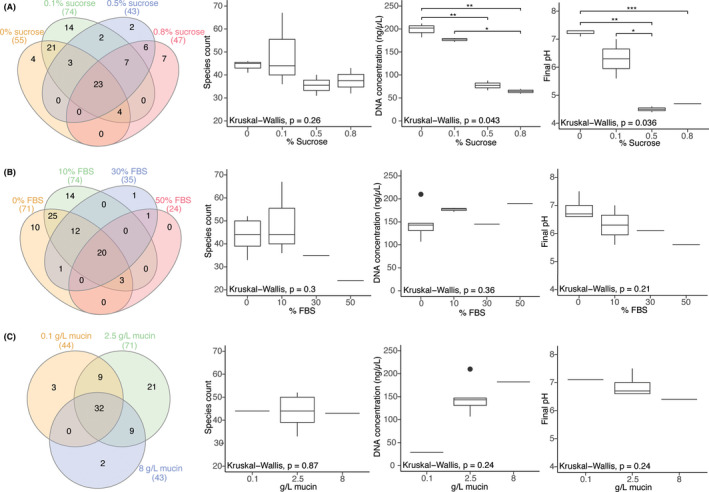
Venn diagram of the number of shared species and boxplots of species count, DNA concentration, and final pH for (A) varying sucrose concentrations with fixed 10% FBS and 2.5g/L mucin; (B) varying FBS concentrations with fixed 0.1% sucrose and 2.5g/L mucin; and (C) varying mucin concentrations with fixed 0.1% sucrose and 0% FBS. If a species was present in at least one replicate, then it was represented in the Venn diagram. Significance is shown with asterisks, all conditions without asterisks are not significant; *: *p* ≤ .05, **: *p* ≤ .01, ***: *p* ≤ .001 [Colour figure can be viewed at wileyonlinelibrary.com]

Starting with SHI medium originally developed to support growth of supragingival microbial communities, adjustments were made to levels of sucrose, FBS, and mucin. Sucrose is the primary carbon source for supragingival, cariogenic organisms, and original SHI medium contains 0.5% sucrose to support high cell density biofilm community growth (Tian et al., 2010). However, subgingival pockets in the oral cavity are less exposed to dietary sugars or the breakdown products from saccharolytic bacteria and the organisms that increase in abundance during the disease state are predominately proteolytic, depending more on peptides for metabolism (Marsh, 2003; Wei et al., 1999). Here, in vitro biofilms were grown in media containing 0%–0.8% sucrose (Table 1). When FBS and mucin were held constant at 10% and 2.5 g/L, respectively, conditions of 0%–0.1% sucrose had significantly (*p* < .05) higher DNA yield, greater number of species (*p* = .09), and significantly (*p* < .05) higher pH compared to biofilms in 0.5%–0.8% sucrose (Figure 2a).

**FIGURE 3:**
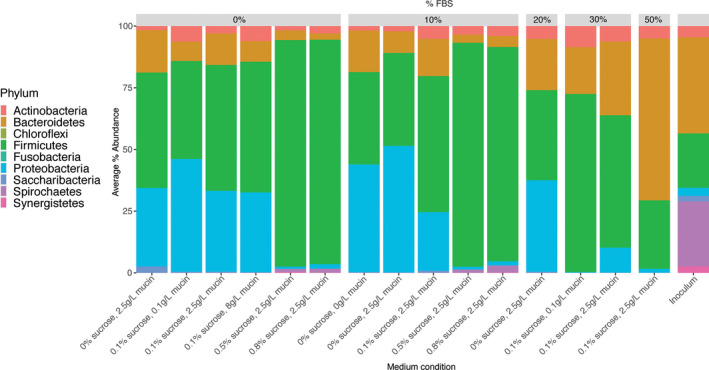
Average percent abundance of phyla within each medium condition and the plaque inoculum. For each condition, ASVs were averaged across replicates before percent abundance was calculated [Colour figure can be viewed at wileyonlinelibrary.com]

At the phyla level, biofilms with 0%–0.1% sucrose were primarily composed of Firmicutes, Proteobacteria, and Bacteroidetes, especially when paired with low or no FBS (Figure 3). These low sucrose conditions were consistently able to capture a majority of phyla found in the inoculum, lacking only Synergistetes and Chloroflexi. Proteobacteria, a phylum that composed only 3% of the inoculum, was greatly enriched in low sucrose conditions, reaching as much as 51% of the in vitro community (Figure 3). When 0.5%–0.8% sucrose was added, Firmicutes increased to about 90% of the community (consisting mostly of the genera *Streptococcus*, *Granulicatella*, and *Gemella*) (Figure S1a).

The change in species composition associated with varying sucrose concentrations (from 0%–0.8% sucrose) was evaluated from biofilms with fixed 10% FBS and 2.5 g/L mucin (Figure 2a; Table S1). Twenty‐three species were found across these sucrose concentrations, including *P. endodontalis*, *Eikenella corrodens*, and several *Streptococcus* and *Actinomyces* species. In conditions with 10% FBS and 2.5 g/L mucin *T. forsythia* was present in samples with 0.1%–0.8% sucrose, although this species was also found in other medium conditions with 0% sucrose (Table S1). Four species were unique to 0% sucrose (including *Fusobacterium nucleatum* subsp. *animalis*), while 14 species were unique to 0.1% sucrose (including seven *Prevotella spp*.). As sucrose concentration increased, 39 species found in 0% and/or 0.1% sucrose conditions were lost from the community, including members of the gram‐negative *Prevotella*, *Campylobacter*, *Dialister*, *Johnsonella, Selenomonas*, the Saccharibacteria bacterium HMT‐351, and several *Streptococcus* and *Parvimonas* species. Conversely, 15 species appeared in the high sucrose conditions that were missing from low sucrose; including the gram‐positive *Lactobacillus*, *Olsenella*, *Peptostreptococcaceae*, and *Rothia*, and members of the gram‐negative *Fretibacterium* and *Treponema*.

### 3.3 FBS variations

Gingival crevicular fluid is a serum exudate that consistently bathes plaque within periodontal pockets and is a protein source for proteolytic taxa within that niche. Inflammation caused by accumulated subgingival plaque leads to an increased flow of GCF, which is correlated with the dysbiotic shift from health to disease (Hajishengallis, 2014). FBS is a common medium supplement that contains similar proteins and growth factors to GCF for subgingival biofilms grown in vitro, but was not a constituent of original SHI medium. In this study, in vitro biofilms were grown in SHI medium with 0%–50% FBS (Table 1). When sucrose and mucin were held constant at 0.1% and 2.5 g/L, respectively, FBS concentration did not affect DNA yield, but low FBS conditions (0%–10%) did have a slightly greater species number (*p* = .13) and higher final pH (*p* = .10) than high FBS conditions (30%–50%) (Figure 2b).

Biofilms with 0%–10% FBS mainly contained the phyla Firmicutes, Proteobacteria, and Bacteroidetes, especially when paired with low or no sucrose (Figure 3). These conditions were consistently able to capture a majority of phyla found in the inoculum, lacking only Synergistetes and Chloroflexi. With the inclusion of additional FBS, Bacteroidetes increased from less than 20% in no FBS conditions to 66% in the 50% FBS condition (Figure 3, Figure S1b) and overall phylum diversity decreased, with the 50% FBS condition containing only four of the nine phyla from the inoculum.

The change in species composition associated with varying FBS concentrations (from 0%–50%) was also evaluated from biofilms with 0.1% sucrose and 2.5 g/L mucin. Twenty species were found across these FBS conditions, including *E. corrodens*, *Haemophilus parainfluenzae*, and several *Actinomyces* and *Streptococcus* species (Figure 2b; Table S1). Ten species were unique to 0% FBS (including *T. denticola* and *F. nucleatum* subsp. *animalis*), while 14 species were unique to 10% FBS including members of the genera *Prevotella*, *Treponema*, and Saccharibacteria (Figure 2b). As FBS concentration increased to 30% and 50%, 49 species found in 0% and/or 10% FBS were lost from the community, including members of the gram‐negative *Alloprevotella*, *Prevotella, Treponema,* and the Saccharibacteria. *Prevotella melaninogenica*, in the phylum Bacteroidetes, became increasingly prevalent as FBS concentration increased. Comprising only 0.5% relative abundance of the inoculum, this species steadily increased from less than 4% in conditions without FBS to half of the resulting culture in the 50% FBS condition.

### 3.4 Mucin variations

Salivary mucins and mucin glycans are a source of carbohydrate associated with the metabolism of supragingival species (Bradshaw et al., 1994; Glenister et al., 1988; Van der Hoeven et al., 1991) and 2.5 g/L mucin is included in the original SHI medium. Relatively little is known about how regulating the abundance of this complex substrate may impact oral community composition and mucins may not be a necessary carbon source for the more asaccharolytic subgingival communities within the periodontal pocket that are less exposed to saliva. In the present study we varied the media with 0–8 g/L mucin. When FBS and sucrose were held constant at 0% and 0.1%, respectively, there was no difference in average species count; however, DNA concentration decreased at lower mucin levels (Figure 2c). Despite there being no average difference in species count between these three mucin concentrations, 21 species were found only in 2.5 g/L mucin conditions (Figure 2c).

The complete removal of mucin was associated with a slight increase in Bacteroidetes (from 9% to 17%) and decrease in Proteobacteria (from 51% to 44%) without changing concentrations of Firmicutes (Figure 3). Firmicutes (specifically *Streptococcus spp*.) were slightly enriched when concentration increased from 0.1 to 8.0 g/L mucin.

### 3.5 Similarity to source plaque and growth of subgingival species

All in vitro biofilms were examined for fidelity to the plaque inoculum in terms of both species proportion and abundance, with specific focus on the enrichment of subgingival species. Of the organisms present in the inoculum, all nine phyla, 43 of the 51 genera, and 89 of the 119 species were captured in at least one in vitro biofilm. Four genera (*Veillonella*, *Lactobacillus*, *Solobacterium*, and *Neisseria*) comprising 34 species were present in some in vitro communities but were not sequenced in the plaque inoculum; these genera were likely present at a low abundance in the inoculum and were therefore not detected via sequencing.

**FIGURE 4:**
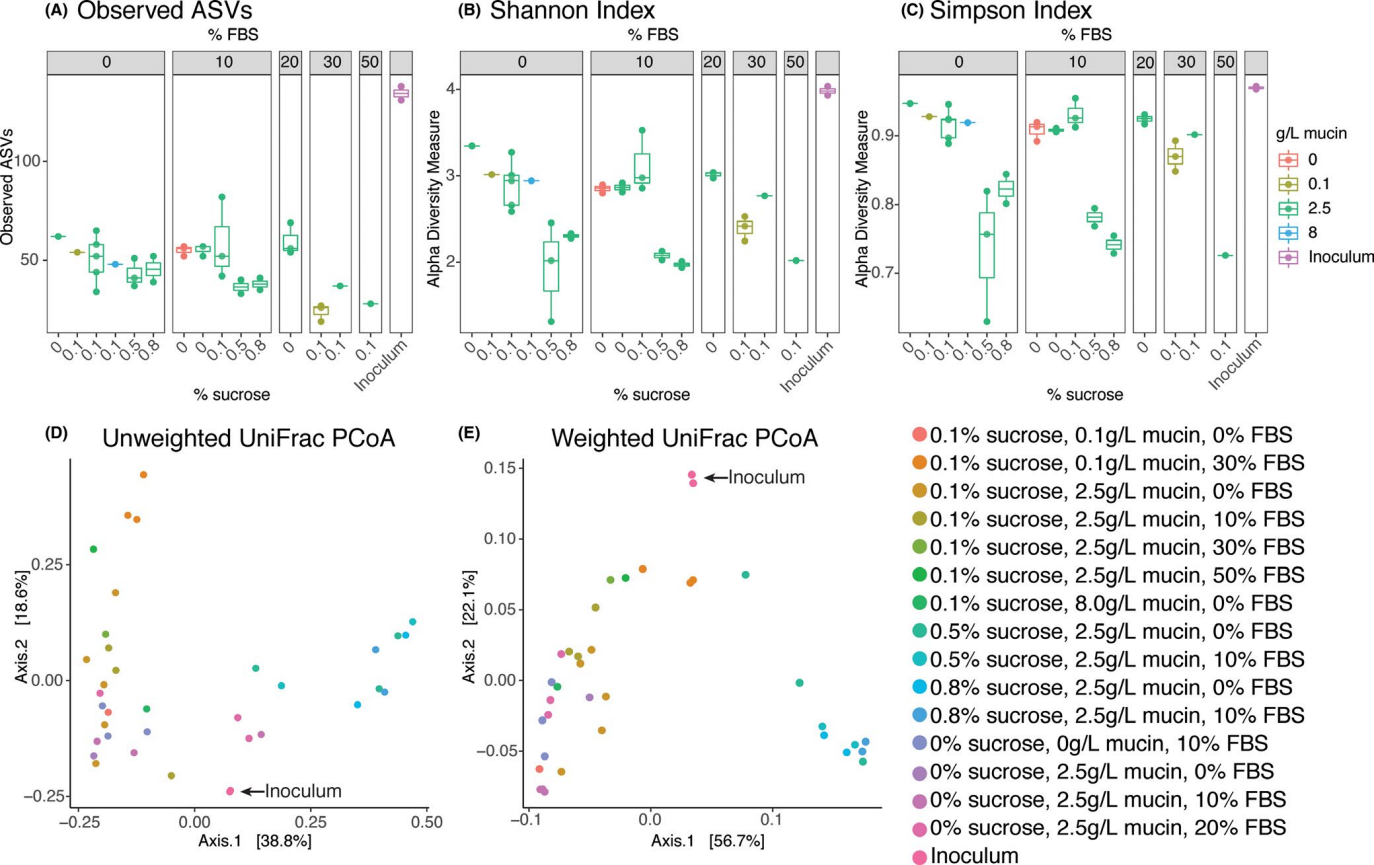
Alpha and Beta diversity analyses for all in vitro biofilms and the plaque inoculum. Alpha diversity was measured with (A) observed ASVs, (B) Shannon indices, and (C) Simpson indices using rarefied data. Beta diversity was measured with (D) Unweighted UniFrac and (E) Weighted UniFrac distances. [Colour figure can be viewed at wileyonlinelibrary.com]

Rarefied measures of alpha diversity (Observed ASVs, Shannon, and Simpson) are presented for each media condition in Figure 4a–c. All in vitro biofilms had similar observed ASV counts, with a mean of 49.2 ASVs ± SD14.0 (*n* = 36). Conditions with high sucrose (0.5%–0.8%) had consistently lower alpha diversity than conditions of low sucrose (0%–0.1%). The high FBS (50%) condition also showed similarly low alpha diversity. Both Shannon and Simpson indices revealed a higher alpha diversity in biofilms with 0%–0.1% sucrose and 0%–20% FBS, regardless of mucin concentration. Beta diversities were measured using unweighted and weighted UniFrac distances and plotted using principal coordinate analyses (PCoA) shown in Figure 4d,e. The similarity in beta diversity between two independent analyses of the plaque inoculum is indicative of reproducible results between sequencing runs. In both PCoAs, the inoculum was positioned in between two separate clusters of low (0%–0.1%) and high (0.5%–0.8%) sucrose.

Beta diversity analyses revealed the conditions that approached the composition of the plaque inoculum. Of the conditions closest to the inoculum on the PCoAs, biofilms grown in media with low (0.1%) sucrose and low (10%) FBS had high alpha diversities and supported communities with taxonomic abundances and proportions most representative of the disease‐state subgingival plaque inoculum (Figures 1c, 3). Mucin did not appear to have a significant impact on the community composition but did increase the overall biomass. At this stage in development, the medium modified from SHI medium formulated to contain 0.1% sucrose, 10% FBS, and 2.5 g/L mucin best captured a subgingival community composition and was given the name subSHI‐v1 medium (subgingival medium version 1). Although similar in diversity metrics to biofilms grown in media with no sucrose or with 30% FBS, the in vitro communities from subSHI‐v1 medium captured a greater number of subgingival species of interest than other media conditions, including members of *Prevotella*, *Treponema*, and the Candidate Phyla Radiation (CPR) Saccharibacteria, most notably the G3 HMT‐351 (“Ca. *N. nanoralicus”*).

**FIGURE 5:**
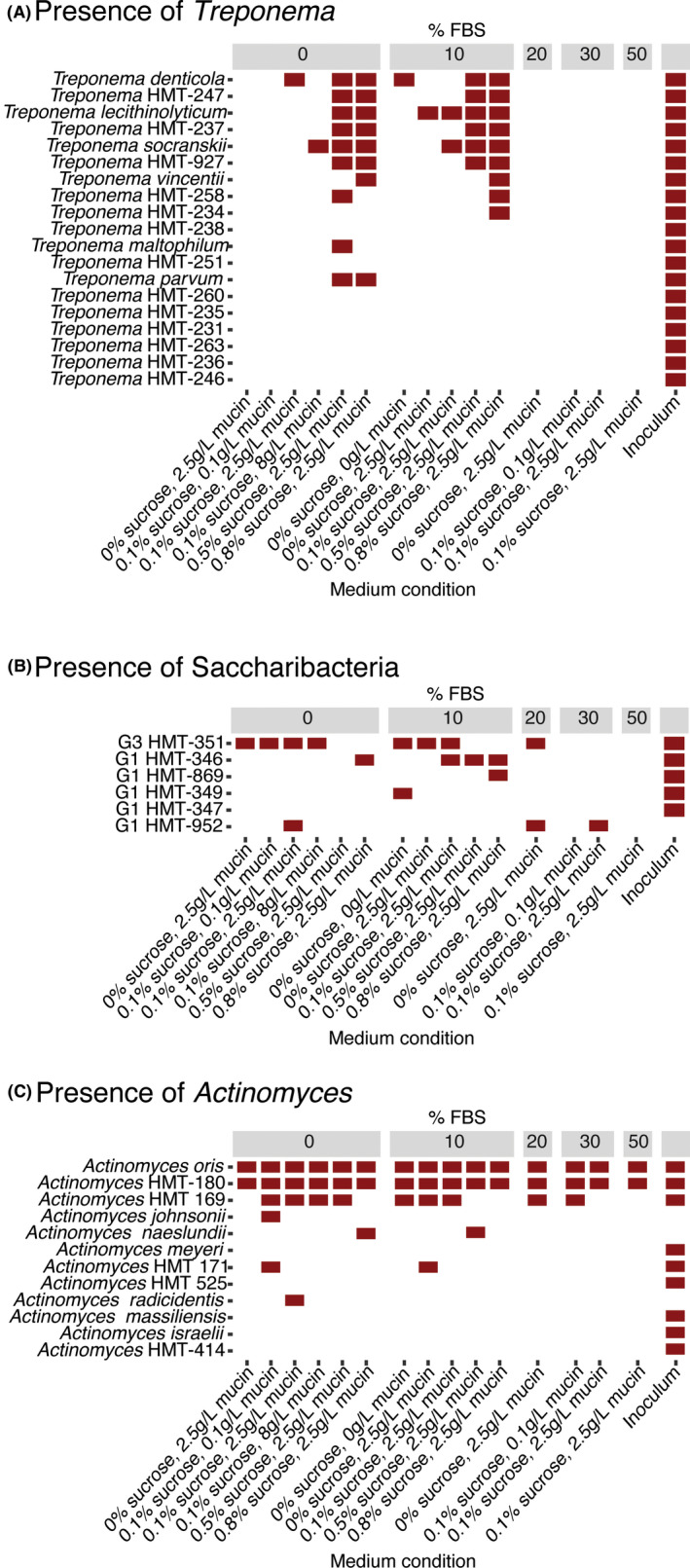
The presence of (A) *Treponema spp.,* (B) Saccharibacteria spp., and (C) *Actinomyces spp*. in each medium condition. A species was considered present in the condition if it was detected in at least one in vitro biofilm replicate [Colour figure can be viewed at wileyonlinelibrary.com]

Despite the relatively higher diversity and inclusion of subgingival species of interest seen in subSHI‐v1 medium, this condition was unable to fully reproduce the high diversity community of the plaque inoculum. Biofilms grown in subSHI‐v1 medium were most notably lacking representatives of the phyla Chloroflexi, Synergistes, and Spirochaetes. While Chloroflexi and Synergistes were at relatively low abundances in the plaque inoculum, the genus *Treponema* within the Spirochaetes constituted 26% of this in vivo community (Figure 1a). No Chloroflexi or Synergistes taxa were present in any low sucrose low FBS condition, and these biofilms contained only one or two members of the *Treponema* (Figure 5a). Unexpectedly, these three phyla were present in high sucrose conditions (Figures 1b, 3). Although Chloroflexi and Synergistes were still at low proportions (averaging 0.0025% and 0.15%, respectively), each of the four high sucrose biofilms captured between six and nine *Treponema* taxa which averaged 1.72% of the in vitro community.

### 3.6 Partial genome of “Ca. *N. nanoralicus*” and potential host

The Saccharibacteria member G3 HMT‐351, comprising 0.2% of the plaque inoculum, was present in multiple media conditions of 0%–20% FBS and 0%–0.1% sucrose (Figure 5b). WGS sequencing was performed on two in vitro biofilms that showed enriched growth of G3 HMT‐351 to extract a partial genome of this strain. The resulting assembly had 39 contigs with a total length of 718,376 bp and an average G + C content of 41.4%. It contained 719 coding sequences (CDS) and 44 RNA genes.

In addition to *N. lyticus* strain TM7x, several cultured representatives of the Saccharibacteria have been isolated recently with hosts from the phylum Actinobacteria (Bor et al., 2020; Cross et al., 2019). Within our in vitro biofilms, *Actinomyces oris*, HMT‐180, and HMT‐169 were present in most or all biofilms containing G3 HMT‐351 (Figure 5c). *A. oris* and HMT‐180 were in all biofilms regardless of G3 HMT‐351 presence, and the average species abundance of *Actinomyces sp*. HMT‐169 was significantly higher (*p* < .05) in biofilms containing G3 HMT‐351. These three *Actinomyces* species therefore represent potential hosts of this reduced genome epibiont.

## 4 DISCUSSION

The shift from oral homeostasis to dysbiosis leads to periodontitis and is influenced by interactions of a diverse microbial community with the host. To carry out in vitro experiments of subgingival health and disease, it is imperative to have a comprehensive lab‐grown biofilm model that emulates site‐specific in vivo communities (including uncultivated taxa) (Colombo & Tanner, 2019; Edlund et al., 2013). In this study, we altered just three major components of SHI medium (which maintains a healthy supragingival in vitro community) to generate subSHI‐v1 medium that now has the capability to accommodate bacteria found within a disease‐state subgingival community.

In vitro biofilms grown in SHI medium with high (0.5%–0.8%) sucrose had low final pH of ~4.6 (Figure 2a) and supported communities composed of over 90% saccharolytic Firmicutes that more closely resembled the supragingival health‐derived communities grown in original SHI medium (Figure 1b; Edlund et al., 2013; Tian et al., 2010). Disease‐associated subgingival bacteria are considered to grow best in alkaline environments (Barros et al., 2016), and this low pH likely limited the growth of subgingival taxa in high sucrose biofilms. Decreasing sucrose to 0%–0.1% dramatically altered microbial composition and supported a diverse community no longer dominated by Firmicutes, showing that a high concentration of sucrose is not necessary for growth of the subgingival microbiota and may even be adverse to the model. When biofilms with varying sucrose conditions were compared, only four species were unique to 0% sucrose; however, they were in few samples and at a low abundance. In contrast, 38 species were only sequenced from sucrose‐containing media (Figure 2b). This result agrees with Baraniya et al. (2020) findings where media containing 0.1% sucrose supported higher growth of subgingival bacteria. The new medium (subSHI‐v1) formulation includes 0.1% sucrose to generate an in vitro community closest to the plaque inoculum.

While not present in the original SHI medium, serum is a common medium supplement that contains similar proteins and nutrients to GCF (Ammann et al., 2012; Hope & Wilson, 2006; ter Steeg et al., 1988). An addition of 10% FBS to SHI medium modified with 0.1% sucrose did not greatly alter biofilm alpha diversity compared to conditions without FBS, while adding 30%–50% FBS actually decreased the phylum level diversity. Lower alpha diversity has been previously noted at higher serum levels (Baraniya et al., 2020), and a recent study comparing plaque growth in media conditions with or without FBS saw only an enrichment of Bacteroidetes in FBS‐supplemented media (Naginyte et al., 2019). Our in vitro models with 10% FBS did, however, maintain the alpha diversity found in 0% FBS and capture a few additional subgingival organisms of interest (e.g., *Prevotella*, *Treponema*, and Saccharibacteria strains). We therefore supplement subSHI‐v1 medium with 10% FBS to increase the number of subgingival species captured.

Although decreasing or even removing mucin from our modified SHI medium did not have a significant effect on community composition, the total biomass was detrimentally impacted (Figure 2c). Preserving the original SHI‐medium mucin concentration of 2.5g/L neither impacted alpha diversity nor resulted in substantial loss of key subgingival species. These preliminary results suggest mucin may not be a necessary component of media for a subgingival model nor do low amounts have a large impact. However, the presence of mucin may be important when generating a temporal model of the transition from health to disease state, and here we keep the same 2.5 g/L mucin concentration in subSHI‐v1 medium.

A major benefit of developing oral communities in vitro is the ability to study fastidious organisms with unknown requirements for growth. The first cultured member of the Saccharibacteria phylum, *N. lyticus* stain TM7x, was isolated from a community grown in original SHI‐medium and determined to be an ultrasmall epibiont on its bacterial host *Actinomyces odontolyticus* (He et al., 2015). Currently, a limited number of Saccharibacteria strains have been isolated on their hosts, mostly Actinobacteria (Bor et al., 2020; Cross et al., 2019). In the current study, the Saccharibacteria member G3 HMT‐351 provisionally given the genus and species name “Ca. *Nanosyncoccus nanoralicus*” (McLean et al., 2018, 2020) was enriched in several low sucrose, low FBS conditions to proportions equal to or greater than that of the inoculum (Figure 5b). “Ca. *N. nanoralicus”* enriched cultures were examined for potential Actinobacteria hosts. *Actinomyces sp*. HMT‐169 was found to be at significantly higher proportions and may be the host of this epibiont. Recently, Cross et al. (2019) maintained a coculture of G3 HMT‐351 on *Actinomyces* sp. HOT‐897; however, this Actinobacterium was not present in our plaque inoculum or in vitro communities. It is possible that “Ca. *N. nanoralicus”* exhibits limited flexibility in its host range as recently demonstrated for TM7x (Bor et al., 2020; Utter et al., 2020).

Members of the gram‐negative genus *Treponema* are of interest as their diversity and abundance correlates with increased severity of disease, and they can comprise up to 50% of an advanced disease community (Armitage et al., 1982; Curtis et al., 2020; Lindhe et al., 1980). At least 50 species‐level taxa of *Treponema* have been sequenced from periodontitis samples, but they have proven difficult to culture in the lab and a large proportion of known taxa are uncultured (Dewhirst et al., 2000, 2010; You et al., 2013). Counterintuitively, high growth of *Treponema* was detected in biofilms with 0.5%–0.8% sucrose (Figure 5a). Several cultured treponemes have the ability to metabolize sugars, including *T. socranskii* that can ferment sucrose (Chan & McLaughlin, 2000; Sakamoto et al., 1999; Smibert et al., 1984; Wyss et al., 2001) and *T. denticola* that can ferment glucose (Hespell & Canale‐Parola, 1971; Tanno‐Nakanishi et al., 2018). The high sucrose environment of these in vitro biofilms could be supporting the metabolism of *Treponema* with different nutritional requirements, possibly consuming either the sucrose itself or the metabolic byproducts of streptococci metabolism. The definitive growth of a diverse array of treponemes in our high sucrose biofilms is an exciting observation that may shine light on the media conditions required to isolate and culture these organisms. Further work is needed to understand how to better maintain in vivo levels of *Treponema* in the in vitro community, which appear to be outcompeted and at odds with the current formulations that capture much of the species in this aggressive periodontitis sample.

Although in vitro communities grown in subSHI‐v1 medium maintained the presence of major phyla from the original inoculum, preserving their relative abundances was more challenging. Over‐enrichment of Firmicutes appears a consistent feature of subgingival biofilm models (Baraniya et al., 2020; Fernandez y Mostajo et al., 2017; Walker & Sedlacek, 2007), and Firmicutes were found in high abundances in almost all of our in vitro biofilms, becoming more dominant as sucrose concentration increased. Bacteroidetes, however, were less abundant in in vitro biofilms, increasing at higher FBS concentrations only with the enrichment of one species *P. melaninogenica* and losing the diversity of the phylum. Another phylum Proteobacteria, comprising only 3% of the plaque inoculum, was enriched up to 50% of the community in low sucrose, low FBS conditions. This is particularly interesting as other recent subgingival models have been unable to capture much Proteobacteria in their communities (Baraniya et al., 2020; Naginyte et al., 2019).

The current study brings us a step closer to a representative subgingival in vitro model. While none of the modified media fully captured the high diversity of the plaque inoculum, our systematic approach to alter a known supragingival growth medium revealed that the new subSHI‐v1 medium with minimal changes to a lower sucrose concentration (0.1% sucrose), the addition of 10% FBS, and preserving the original 2.5 g/L mucin concentration was most effective in approaching both the overall diversity and the gram‐negative subgingival species that were present in the diseased plaque. The details presented here on community composition of biofilms grown in other SHI media formulations can additionally aid researchers varying media to reach their own research goals, which may include enriching for different species of interest. Although only one periodontitis sample was used to refine subSHI‐v1 medium, we demonstrate that in vitro biofilm growth from a single patient can reveal features of individual periodontitis sites. We anticipate this model may be able to capture more of the diversity found in other site‐specific subgingival samples and will increase our knowledge of the transition between a healthy, gram‐positive bacteria dominated plaque community to a gram‐negative rich community.

## CONFLICT OF INTERESTS

The authors have no conflict of interests to declare.

### PEER REVIEW

The peer review history for this article is available at https://publons.com/publon/10.1111/omi.12323.

## Supporting information

Fig S1

Table S1

## References

[omi12323-bib-0001] Ammann, T. W. , Gmür, R. , & Thurnheer, T. (2012). Advancement of the 10‐species subgingival Zurich biofilm model by examining different nutritional conditions and defining the structure of the in vitro biofilms. BMC Microbiology, 12. 10.1186/1471-2180-12-227 PMC3561252 23040057

[omi12323-bib-0002] Andersen, K. S. , Kirkegaard, R. H. , Karst, S. M. , & Albertsen, M. (2018). ampvis2: An R package to analyse and visualise 16S rRNA amplicon data. BioRxiv (Preprint), 299537. 10.1101/299537

[omi12323-bib-0003] Arkin, A. P. , Cottingham, R. W. , Henry, C. S. , Harris, N. L. , Stevens, R. L. , Maslov, S. , Dehal, P. , Ware, D. , Perez, F. , Canon, S. , Sneddon, M. W. , Henderson, M. L. , Riehl, W. J. , Murphy‐Olson, D. , Chan, S. Y. , Kamimura, R. T. , Kumari, S. , Drake, M. M. , Brettin, T. S. , … Yu, D. (2018). July 6). KBase: The United States department of energy systems biology knowledgebase. Nature Biotechnology, 36, 566–569. 10.1038/nbt.4163 PMC6870991 29979655

[omi12323-bib-0004] Armitage, G. C. (2010). Comparison of the microbiological features of chronic and aggressive periodontitis. Periodontology 2000, 53(1), 70–88. 10.1111/j.1600-0757.2010.00357.x 20403106

[omi12323-bib-0005] Armitage, G. C. , Dickinson, W. R. , Jenderseck, R. S. , Levine, S. M. , & Chambers, D. W. (1982). Relationship between the percentage of subgingival spirochetes and the severity of periodontal disease. Journal of Periodontology, 53(9), 550–556. 10.1902/jop.1982.53.9.550 6957592

[omi12323-bib-0006] Baraniya, D. , Naginyte, M. , Chen, T. , Albandar, J. M. , Chialastri, S. M. , Devine, D. A. , Marsh, P. D. , & Al‐hebshi, N. N. (2020). Modeling normal and dysbiotic subgingival microbiomes: Effect of nutrients. Journal of Dental Research, 99(6), 695–702. 10.1177/0022034520902452 31999932 PMC7243421

[omi12323-bib-0007] Barros, S. P. , Williams, R. , Offenbacher, S. , & Morelli, T. (2016). Gingival crevicular fluid as a source of biomarkers for periodontitis. Periodontology 2000, 70(1), 53–64. 10.1111/prd.12107 26662482 PMC4911175

[omi12323-bib-0008] Bor, B. , Collins, A. J. , Murugkar, P. P. , Balasubramanian, S. , To, T. T. , Hendrickson, E. L. , Bedree, J. K. , Bidlack, F. B. , Johnston, C. D. , Shi, W. , McLean, J. S. , He, X. , & Dewhirst, F. E. (2020). Insights obtained by culturing saccharibacteria with their bacterial hosts. Journal of Dental Research, 99(6), 685–694. 10.1177/0022034520905792 32075512 PMC7243422

[omi12323-bib-0009] Bradshaw, D. J. , Homer, K. A. , Marsh, P. D. , & Beighton, D. (1994). Metabolic cooperation in oral microbial communities during growth on mucin. Microbiology, 140(12), 3407–3412. 10.1099/13500872-140-12-3407 7881558

[omi12323-bib-0010] Callahan, B. J. , McMurdie, P. J. , Rosen, M. J. , Han, A. W. , Johnson, A. J. A. , & Holmes, S. P. (2016). DADA2: High‐resolution sample inference from Illumina amplicon data. Nature Methods, 13(7), 581–583. 10.1038/nmeth.3869 27214047 PMC4927377

[omi12323-bib-0011] Chan, E. C. S. , & McLaughlin, R. (2000). Taxonomy and virulence of oral spirochetes. Oral Microbiology and Immunology, 15, 1–9. 10.1034/j.1399-302X.2000.150101.x 11155157

[omi12323-bib-0012] Cieplik, F. , Zaura, E. , Brandt, B. W. , Buijs, M. J. , Buchalla, W. , Crielaard, W. , Laine, M. L. , Deng, D. M. , & Exterkate, R. A. M. (2019). Microcosm biofilms cultured from different oral niches in periodontitis patients. Journal of Oral Microbiology, 11(1), 1551596. 10.1080/20022727.2018.1551596 30598734 PMC6263112

[omi12323-bib-0013] Colombo, A. P. V. , & Tanner, A. C. R. (2019). The role of bacterial biofilms in dental caries and periodontal and peri‐implant diseases: A historical perspective. Journal of Dental Research, 98(4), 373–385. 10.1177/0022034519830686 30890060

[omi12323-bib-0014] Cross, K. L. , Campbell, J. H. , Balachandran, M. , Campbell, A. G. , Cooper, S. J. , Griffen, A. , Heaton, M. , Joshi, S. , Klingeman, D. , Leys, E. , Yang, Z. , Parks, J. M. , & Podar, M. (2019). Targeted isolation and cultivation of uncultivated bacteria by reverse genomics. Nature Biotechnology, 37(11), 1314–1321. 10.1038/s41587-019-0260-6 PMC6858544 31570900

[omi12323-bib-0015] Curtis, M. A. , Diaz, P. I. , & Van Dyke, T. E. (2020). The role of the microbiota in periodontal disease. Periodontology 2000, 83(1), 14–25. 10.1111/prd.12296 32385883

[omi12323-bib-0016] Dewhirst, F. E. , Chen, T. , Izard, J. , Paster, B. J. , Tanner, A. C. R. , Yu, W.‐H. , Lakshmanan, A. , & Wade, W. G. (2010). The human oral microbiome. Journal of Bacteriology, 192(19), 5002–5017. 10.1128/JB.00542-10 20656903 PMC2944498

[omi12323-bib-0017] Dewhirst, F. E. , Tamer, M. A. , Ericson, R. E. , Lau, C. N. , Levanos, V. A. , Boches, S. K. , Galvin, J. L. , & Paster, B. J. (2000). The diversity of periodontal spirochetes by 16S rRNA analysis. Oral Microbiology and Immunology, 15(3), 196–202. 10.1034/j.1399-302X.2000.150308.x 11154403

[omi12323-bib-0018] Dzink, J. L. , Tanner, A. C. R. , Haffajee, A. D. , & Socransky, S. S. (1985). Gram negative species associated with active destructive periodontal lesions. Journal of Clinical Periodontology, 12(8), 648–659. 10.1111/j.1600-051X.1985.tb00936.x 3863838

[omi12323-bib-0019] Edlund, A. , Yang, Y. , Hall, A. P. , Guo, L. , Lux, R. , He, X. , Nelson, K. E. , Nealson, K. H. , Yooseph, S. , Shi, W. , & McLean, J. S. (2013). An in vitro biofilm model system maintaining a highly reproducible species and metabolic diversity approaching that of the human oral microbiome. Microbiome, 1(1), 1–17. 10.1186/2049-2618-1-25 24451062 PMC3971625

[omi12323-bib-0020] Edlund, A. , Yang, Y. , Yooseph, S. , Hall, A. P. , Nguyen, D. D. , Dorrestein, P. C. , Nelson, K. E. , He, X. , Lux, R. , Shi, W. , & McLean, J. S. (2015). Meta‐omics uncover temporal regulation of pathways across oral microbiome genera during in vitro sugar metabolism. The ISME Journal, 9(12), 2605–2619. 10.1038/ismej.2015.72 26023872 PMC4817640

[omi12323-bib-0021] Edlund, A. , Yang, Y. , Yooseph, S. , He, X. , Shi, W. , & McLean, J. S. (2018). Uncovering complex microbiome activities via metatranscriptomics during 24 hours of oral biofilm assembly and maturation. Microbiome, 6(1), 1–22. 10.1186/s40168-018-0591-4 30522530 PMC6284299

[omi12323-bib-0022] Eke, P. I. , Thornton‐Evans, G. O. , Wei, L. , Borgnakke, W. S. , Dye, B. A. , & Genco, R. J. (2018). Periodontitis in US adults: National Health and Nutrition Examination Survey 2009–2014. Journal of the American Dental Association, 149(7), 576–588.e6. 10.1016/j.adaj.2018.04.023 29957185 PMC8094373

[omi12323-bib-0023] Escapa, I. F. , Chen, T. , Huang, Y. , Gajare, P. , Dewhirst, F. E. , & Lemon, K. P. (2018). New insights into human nostril microbiome from the Expanded Human Oral Microbiome Database (eHOMD): A resource for the microbiome of the human aerodigestive tract. Msystems, 3(6). 10.1128/msystems.00187-18 e00187–18. PMC6280432 30534599

[omi12323-bib-0024] Fernandez y Mostajo, M. , Exterkate, R. A. M. , Buijs, M. J. , Beertsen, W. , van der Weijden, G. A. , Zaura, E. , & Crielaard, W. (2017). A reproducible microcosm biofilm model of subgingival microbial communities. Journal of Periodontal Research, 52(6), 1021–1031. 10.1111/jre.12473 28707424

[omi12323-bib-0025] Glenister, D. A. , Salamon, K. E. , Smith, K. , Beighton, D. , & Keevil, C. W. (1988). Enhanced growth of complex communities of dental plaque bacteria in mucin‐limited continuous culture. Microbial Ecology in Health and Disease, 1(1), 31–38. 10.3109/08910608809140176

[omi12323-bib-0026] Hajishengallis, G. (2014). The inflammophilic character of the periodontitis‐associated microbiota. Molecular Oral Microbiology, 29(6), 248–257. 10.1111/omi.12065 24976068 PMC4232466

[omi12323-bib-0027] He, X. , McLean, J. S. , Edlund, A. , Yooseph, S. , Hall, A. P. , Liu, S.‐Y. , Dorrestein, P. C. , Esquenazi, E. , Hunter, R. C. , Cheng, G. , Nelson, K. E. , Lux, R. , & Shi, W. (2015). Cultivation of a human‐associated TM7 phylotype reveals a reduced genome and epibiotic parasitic lifestyle. Proceedings of the National Academy of Sciences of the United States of America, 112(1), 244–249. 10.1073/pnas.1419038112 25535390 PMC4291631

[omi12323-bib-0028] Heberle, H. , Meirelles, V. G. , da Silva, F. R. , Telles, G. P. , & Minghim, R. (2015). InteractiVenn: A web‐based tool for the analysis of sets through Venn diagrams. BMC Bioinformatics, 16(1), 169. 10.1186/s12859-015-0611-3 25994840 PMC4455604

[omi12323-bib-0029] Hespell, R. B. , & Canale‐Parola, E. (1971). Amino acid and glucose fermentation by Treponema denticola. Archiv Für Mikrobiologie, 78(3), 234–251. 10.1007/BF00424897 4938689

[omi12323-bib-0030] Hope, C. K. , & Wilson, M. (2006). Biofilm structure and cell vitality in a laboratory model of subgingival plaque. Journal of Microbiological Methods, 66(3), 390–398. 10.1016/j.mimet.2006.01.003 16487610

[omi12323-bib-0031] Lamont, R. J. , Koo, H. , & Hajishengallis, G. (2018). The oral microbiota: Dynamic communities and host interactions. Nature Reviews Microbiology, 16, 745–759. 10.1038/s41579-018-0089-x 30301974 PMC6278837

[omi12323-bib-0032] Lindhe, J. , Liljenberg, B. , & Listgarten, M. (1980). Some microbiological and histopathological features of periodontal disease in man. Journal of Periodontology, 51(5), 264–269. 10.1902/jop.1980.51.5.264 6929912

[omi12323-bib-0033] Mark Welch, J. L. , Dewhirst, F. E. , & Borisy, G. G. (2019). Biogeography of the oral microbiome: The site‐specialist hypothesis. Annual Review of Microbiology, 73, 335–358. 10.1146/annurev-micro-090817-062503 PMC7153577 31180804

[omi12323-bib-0034] Marsh, P. D. (2003). Are dental diseases examples of ecological catastrophes? Microbiology, 149, 279–294. 10.1099/mic.0.26082-0 12624191

[omi12323-bib-0035] McLean, J. S. (2014). Advancements toward a systems level understanding of the human oral microbiome. Frontiers in Cellular and Infection Microbiology, 4, 98. 10.3389/fcimb.2014.00098 25120956 PMC4114298

[omi12323-bib-0062] McMurdie, P. J. , & Holmes, S. (2013). phyloseq: an R package for reproducible interactive analysis and graphics of microbiome census data. PLoS One, 8(4), e61217 10.1371/journal.pone.0061217. 23630581 PMC3632530

[omi12323-bib-0036] McLean, J. S. , Bor, B. , Kerns, K. A. , Liu, Q. , To, T. T. , Solden, L. , Hendrickson, E. L. , Wrighton, K. , Shi, W. , & He, X. (2020). Acquisition and adaptation of ultra‐small parasitic reduced genome bacteria to mammalian hosts. Cell Reports, 32(3), 107939. 10.1016/j.celrep.2020.107939 32698001 PMC7427843

[omi12323-bib-0037] McLean, J. S. , Bor, B. , To, T. T. , Liu, Q. , Kerns, K. A. , Solden, L. , & Shi, W. (2018). Evidence of independent acquisition and adaption of ultra‐small bacteria to human hosts across the highly diverse yet reduced genomes of the phylum Saccharibacteria. BioRxiv, 258137. 10.1101/258137

[omi12323-bib-0038] Moore, W. E. , Holdeman, L. V. , Cato, E. P. , Good, I. J. , Smith, E. P. , Ranney, R. R. , & Palcanis, K. G. (1984). Variation in periodontal floras. Infection and Immunity, 46(3), 720–726. 10.1128/IAI.46.3.720-726.1984 6500707 PMC261604

[omi12323-bib-0039] Mountcastle, S. E. , Cox, S. C. , Sammons, R. L. , Jabbari, S. , Shelton, R. M. , & Kuehne, S. A. (2020). A review of co‐culture models to study the oral microenvironment and disease. Journal of Oral Microbiology, 12(1), 1773122. 10.1080/20002297.2020.1773122 32922679 PMC7448840

[omi12323-bib-0040] Naginyte, M. , Do, T. , Meade, J. , Devine, D. A. , & Marsh, P. D. (2019). Enrichment of periodontal pathogens from the biofilms of healthy adults. Scientific Reports, 9(1), 1–9. 10.1038/s41598-019-41882-y 30940882 PMC6445289

[omi12323-bib-0041] Ondov, B. D. , Bergman, N. H. , & Phillippy, A. M. (2011). Interactive metagenomic visualization in a Web browser. BMC Bioinformatics, 12. 10.1186/1471-2105-12-385 385 21961884 PMC3190407

[omi12323-bib-0042] Papapanou, P. N. , Sanz, M. , Buduneli, N. , Dietrich, T. , Feres, M. , Fine, D. H. , Flemmig, T. F. , Garcia, R. , Giannobile, W. V. , Graziani, F. , Greenwell, H. , Herrera, D. , Kao, R. T. , Kebschull, M. , Kinane, D. F. , Kirkwood, K. L. , Kocher, T. , Kornman, K. S. , Kumar, P. S. , … Tonetti, M. S. (2018). Periodontitis: Consensus report of workgroup 2 of the 2017 World Workshop on the Classification of Periodontal and Peri‐Implant Diseases and Conditions. Journal of Periodontology, 89, S173–S182. 10.1002/JPER.17-0721 29926951

[omi12323-bib-0043] Sakamoto, M. , Koseki, T. , Umeda, M. , Ishikawa, I. , Benno, Y. , & Nakase, T. (1999). Phylogenetic analysis of saccharolytic oral treponemes isolated from human subgingival plaque. Microbiology and Immunology, 43(7), 711–716. 10.1111/j.1348-0421.1999.tb02460.x 10529113

[omi12323-bib-0044] Sánchez, M. C. , Llama‐Palacios, A. , Blanc, V. , León, R. , Herrera, D. , & Sanz, M. (2011). Structure, viability and bacterial kinetics of an in vitro biofilm model using six bacteria from the subgingival microbiota. Journal of Periodontal Research, 46(2), 252–260. 10.1111/j.1600-0765.2010.01341.x 21261622

[omi12323-bib-0045] Sissons, C. H. (1997). Artificial dental plaque biofilm model systems. Advances in Dental Research, 11(1), 110–126. 10.1177/08959374970110010201 9524448

[omi12323-bib-0046] Smibert, R. M. , Johnson, J. L. , & Ranney, R. R. (1984). Treponema socranskii sp. nov., Treponema socranskii subsp. socranskii subsp. nov., Treponema socranskii subsp. buccale subsp. nov., and Treponema socranskii subsp. paredis subsp. nov. isolated from the human periodontia. International Journal of Systematic Bacteriology, 34(4), 457–462. 10.1099/00207713-34-4-457

[omi12323-bib-0047] Soares, G. M. S. , Teles, F. , Starr, J. R. , Feres, M. , Patel, M. , Martin, L. , & Teles, R. (2015). Effects of azithromycin, metronidazole, amoxicillin, and metronidazole plus amoxicillin on an in vitro polymicrobial subgingival biofilm model. Antimicrobial Agents and Chemotherapy, 59(5), 2791–2798. 10.1128/AAC.04974-14 25733510 PMC4394767

[omi12323-bib-0048] Socransky, S. S. , Haffajee, A. D. , Cugini, M. A. , Smith, C. , & Kent, R. L. (1998). Microbial complexes in subgingival plaque. Journal of Clinical Periodontology, 25(2), 134–144. 10.1111/j.1600-051X.1998.tb02419.x 9495612

[omi12323-bib-0049] Tanno‐Nakanishi, M. , Kikuchi, Y. , Kokubu, E. , Yamada, S. , & Ishihara, K. (2018). Treponema denticola transcriptional profiles in serum‐restricted conditions. FEMS Microbiology Letters, 365(16). 10.1093/femsle/fny171 1–7. 29982599

[omi12323-bib-0050] Ter Steeg, P. F. , Van Der Hoeven, J. S. , De Jong, M. H. , Van Munster, P. J. J. , Jansen, M. J. H. , Ter Steeg, P. F. , & Jansenf, M. J. H. (1988). Modelling the gingival pocket by enrichment of subgingival microflora in human serum in chemostats. Microbial Ecology in Health and Disease, 1(2), 988. 10.3109/08910608809140185

[omi12323-bib-0051] Theilade, E. (1986). The non‐specific theory in microbial etiology of inflammatory periodontal diseases. Journal of Clinical Periodontology, 13(10), 905–911. 10.1111/j.1600-051X.1986.tb01425.x 3540019

[omi12323-bib-0052] Thompson, H. , Rybalka, A. , Moazzez, R. , Dewhirst, F. E. , & Wade, W. G. (2015). In vitro culture of previously uncultured oral bacterial phylotypes. Applied and Environmental Microbiology, 81(24), 8307–8314. 10.1128/AEM.02156-15 26407883 PMC4644652

[omi12323-bib-0053] Tian, Y. , He, X. , Torralba, M. , Yooseph, S. , Nelson, K. E. , Lux, R. , McLean, J. S. , Yu, G. , & Shi, W. (2010). Using DGGE profiling to develop a novel culture medium suitable for oral microbial communities. Molecular Oral Microbiology, 25(5), 357–367. 10.1111/j.2041-1014.2010.00585.x 20883224 PMC2951289

[omi12323-bib-0054] Utter, D. R. , He, X. , Cavanaugh, C. M. , McLean, J. S. , & Bor, B. (2020). The saccharibacterium TM7x elicits differential responses across its host range. The ISME Journal, 14(12), 3054–3067. 10.1038/s41396-020-00736-6 32839546 PMC7784981

[omi12323-bib-0055] Van der Hoeven, J. , Camp, P. , & Van Der Hoeven, J. (1991). Chemostat cultures synergistic degradation of mucin by streptococcus oralis and streptococcus sanguis in mixed synergistic degradation of mucin by streptococcus oralis and streptococcus sanguis in mixed chemostat cultures. Journal of Dental Research, 70(7), 1041–1044. 10.1177/00220345910700070401 2066484

[omi12323-bib-0056] Velsko, I. M. , & Shaddox, L. M. (2018). Consistent and reproducible long‐term in vitro growth of health and disease‐associated oral subgingival biofilms. BMC Microbiology, 18(1), 70. 10.1186/s12866-018-1212-x 29996764 PMC6042318

[omi12323-bib-0057] Walker, C. , & Sedlacek, M. J. (2007). An in vitro biofilm model of subgingival plaque. Oral Microbiology and Immunology, 22(3), 152–161. 10.1111/j.1399-302X.2007.00336.x 17488440 PMC2020808

[omi12323-bib-0058] Wei, G. X. , Van Der Hoeven, J. S. , Smalley, J. W. , Mikx, F. H. M. , & Fan, M. W. (1999). Proteolysis and utilization of albumin by enrichment cultures of subgingival microbiota. Oral Microbiology and Immunology, 14(6), 348–351. 10.1034/j.1399-302X.1999.140603.x 10895689

[omi12323-bib-0059] Wickham, H. (2016). ggplot2: Elegant Graphics for Data Analysis. https://ggplot2.tidyverse.org

[omi12323-bib-0060] Wyss, C. , Dewhirst, F. E. , Gmür, R. , Thurnheer, T. , Xue, Y. , Schüpbach, P. , & Paster, B. J. (2001). Treponema parvum sp. nov., a small, glucuronic or galacturonic acid‐dependent oral spirochaete from lesions of human periodontitis and acute necrotizing ulcerative gingivitis. International Journal of Systematic and Evolutionary Microbiology, 51(3), 955–962. 10.1099/00207713-51-3-955 11411720

[omi12323-bib-0061] You, M. , Mo, S. , Leung, W. K. , & Watt, R. M. (2013). Comparative analysis of oral treponemes associated with periodontal health and disease. BMC Infectious Diseases, 13(1), 1–13. 10.1186/1471-2334-13-174 23578286 PMC3637317

