# Supplementary material for: Modified SHI medium supports growth of a disease‐state subgingival polymicrobial community in vitro
Source: Mol Oral Microbiol. 2020 Dec 3;36(1):37–49. doi: 10.1111/omi.12323 (PMC7984074; doi:10.1111/omi.12323)
Supplement: Supplementary file 1 — Fig S1 [file OMI-36-37-s001.pdf]

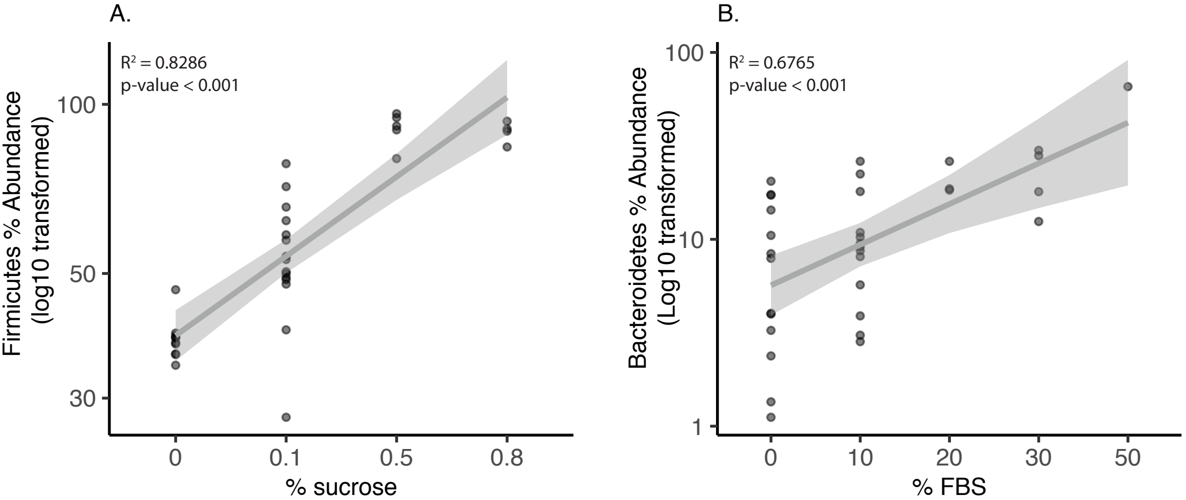

Figure S1 Scatterplot with linear regression and 95% confidence interval showing change in the percent abundance of (A) Firmicutes with increasing sucrose concentration and (B) Bacteroidetes with increasing fetal bovine serum (FBS) concentration for all biofilm samples.
